# Pharmacogenetic Associations of MMP9 and MMP12 Variants with Cardiovascular Disease in Patients with Hypertension

**DOI:** 10.1371/journal.pone.0023609

**Published:** 2011-08-24

**Authors:** Rikki M. Tanner, Amy I. Lynch, Victoria H. Brophy, John H. Eckfeldt, Barry R. Davis, Charles E. Ford, Eric Boerwinkle, Donna K. Arnett

**Affiliations:** 1 Department of Epidemiology, University of Alabama at Birmingham, Birmingham, Alabama, United States of America; 2 Department of Laboratory Medicine and Pathology, University of Minnesota, Minneapolis, Minnesota, United States of America; 3 Department of Human Genetics, Roche Molecular Systems, Pleasanton, California, United States of America; 4 School of Public Health, University of Texas at Houston, Houston, Texas, United States of America; 5 Human Genetics Center, University of Texas at Houston, Houston, Texas, United States of America; Innsbruck Medical University, Austria

## Abstract

**Objectives:**

MMP-9 and -12 function in tissue remodeling and may play roles in cardiovascular disease (CVD). We assessed associations of four MMP polymorphisms and three antihypertensive drugs with cardiovascular outcomes.

**Methods:**

Hypertensives (n = 42,418) from a double-blind, randomized, clinical trial were randomized to chlorthalidone, amlodipine, lisinopril, or doxazosin treatment (mean follow up, 4.9 years). The primary outcome was coronary heart disease (CHD). Secondary outcomes included combined CHD, all CVD outcomes combined, stroke, heart failure (HF), and mortality. Genotype-treatment interactions were tested.

**Results:**

There were 38,698 participants genotyped for at least one of the polymorphisms included here. For *MMP9* R668Q (rs2274756), lower hazard ratios (HRs) were found for AA subjects for most outcomes when treated with chlorthalidone versus amlodipine (eg., CCHD: GG = 1.00, GA = 1.01, AA = 0.64; *P* = 0.038). For *MMP9* R279Q (rs17576), modest pharmacogenetic findings were observed for combined CHD and the composite CVD outcome. For *MMP12* N122S (rs652438), lower HRs were observed for CHD in subjects carrying at least one G allele and being treated with chlorthalidone versus lisinopril (CHD: AA = 1.07, AG = 0.80, GG = 0.49; *P* = 0.005). In the lisinopril-amlodipine comparison, higher HRs were observed for participants having at least one G allele at the *MMP12* N122S locus (CHD: AA = 0.94, AG = 1.19, GG = 1.93; *P* = 0.041). For *MMP12* −82A>G (rs2276109), no pharmacogenetic effect was found for the primary outcome, although lower HRs were observed for AA homozygotes in the chlorthalidone-amlodipine comparison for HF (*P* = 0.015).

**Conclusions:**

We observed interactions between antihypertensive drugs and *MMP9* and *MMP12* for CHD and composite CVD. The data suggest that these genes may provide useful clinical information with respect to treatment decisions.

## Introduction

Matrix metalloproteinases (MMPs) are zinc-dependent endopeptidases which play a role in connective tissue remodeling. Circulating levels of MMPs are associated with many cardiovascular diseases (CVDs), including atherosclerosis, myocardial infarction (MI), and heart failure [1], [2], [3]. MMP-9 (gelatinase-B) functions in the degradation of type IV and V collagens, and its levels are raised in individuals with hypertension, acute coronary syndrome, and acute MI [2], [4], [5]. MMP-12, (macrophage metalloelastase) is up-regulated in atherosclerotic lesions and aneurysms and may contribute to the activation of other MMPs, which, in turn, degrade other extracellular matrix proteins [6]. A recent study found that MMP-12 production by macrophages plays a role in the transition from fatty acids to fibrous plaques during the progression of atherosclerosis [7]. Given MMPs' associations and possible causal connections with CVD, these enzymes represent potential drug targets for CVD treatment and prevention. In fact, amlodipine and lercanidipine have been shown to influence MMP-9 plasma levels and activity [8], [9], [10]. ACE inhibitors may also affect MMP levels [11], [12], [13], [14]. Attempts have been made to assess associations of MMP gene variants with disease and risk phenotypes [15], [16], [17], [18], [19]. For example, individuals carrying the *MMP9* 279Q allele had higher plasma levels of MMP-9 and higher risk of cardiovascular events than patients homozygous for the 279R allele (*P* = 0.02) [16]. The *MMP9* R279Q polymorphism is a glutamine to arginine substitution located in the catalytic domain of MMP-9 [16], [20] and plausibly represents a loss-of-function mutation [21]. The R668Q variant of *MMP9* lies within exon 12. This missense mutation lies in the hemopexin-like domain and probably functions in substrate binding [19]. The *MMP12* −82A>G variant, which lies in the gene's promoter region, affects transcription factor binding affinity, with the A allele associated with increased promoter activity [15], [18]; this variant has been shown to affect coronary artery luminal diameter [15]. The *MMP12* N122S variant lies within the gene's coding region; although this variant has been associated with endpoints such as breast cancer prognosis [22] and diabetic nephropathy [23], the functional significance of the single-nucleotide polymorphism (SNP) is unknown. Given these and other MMP genetic associations with cardiovascular phenotypes and known molecular interactions between antihypertensive drugs and some MMPs, MMP genes may modify rates of CVD outcomes differently for different antihypertensive treatments. We tested whether hypertensive participants in the Genetics of Hypertension Associated Treatment (GenHAT) Study genotyped for *MMP9* variants R279Q and R668Q and for *MMP12* variants N122S and −82A>G and randomized to either the diuretic chlorthalidone, the calcium channel blocker amlodipine (CCB), or the angiotensin converting enzyme (ACE) inhibitor lisinopril had different outcome rates with regard to six CVD phenotypes. (The doxazosin treatment arm was not included in the pharmacogenetic analyses. See below.) All three of these drugs are known to effectively lower blood pressure and all three are commonly prescribed. The effect of treatment assignment on CVD outcomes has been previously published [24], [25]. We sought to determine whether genotype for these variants interacted with treatment assignment to produce a pharmacogenetic association with CVD outcomes.

## Methods

### Ethics statement

Participants recruited during the parent Antihypertensive and Lipid-Lowering Treatment to Prevent Heart Attack Trial (ALLHAT) signed informed consent documents; the GenHAT study was approved by the University of Minnesota Institutional Review Board, the University of Alabama at Birmingham Institutional Review Board for Human Use, and the University of Texas Health Science Center at Houston Committee for the Protection of Human Subjects.

### Study Population

Study participants were part of the GenHAT study, the primary objective of which was to determine whether variants in hypertension susceptibility genes interact with antihypertensive drugs to modify coronary heart disease risk in hypertensive patients [26]. GenHAT is ancillary to the ALLHAT study, a randomized, double-blind clinical trial examining 42,418 hypertensive patients age 55 or older (47% non-White and 46% female) with one or more risk factors for CVD. Participants were randomized to one of four ALLHAT treatment arms—chlorthalidone, lisinopril, amlodipine, and doxazosin, in a ratio of 1.7∶1∶1∶1, respectively [26]. Due to early discontinuation of the doxazosin arm of ALLHAT [27]—and thus fewer clinical outcomes in genotype and treatment groups—we have not included the doxazosin group in these analyses. The drug doses were titrated to achieve blood pressures lower than 140/90 mmHg. Detailed descriptions of both ALLHAT and GenHAT have been published elsewhere [26], [28].

GenHAT genotyped variants in several hypertension-related genes in the 39,114 ALLHAT participants with available DNA, making the study design a post-hoc subgroup analysis of a randomized clinical trial [29]. Participants were excluded from analysis if they were missing genotype data (n = 489 for *MMP9* R279Q, n = 499 for *MMP9* R668Q, n = 1019 for *MMP12* N122S, n = 577 for *MMP12* −82A>G). There were 38,698 participants successfully genotyped for at least one of the polymorphisms included in this analysis.

### Outcomes

The primary outcome for both ALLHAT and GenHAT was coronary heart disease (CHD), defined as fatal CHD or nonfatal myocardial infarction (MI). Secondary outcomes for this study included all-cause mortality (ACM); stroke; chronic heart failure (CHF); combined CHD outcomes (CCHD, defined as CHD death or non-fatal MI plus revascularization procedures plus hospitalized angina); and combined CVD (CCVD, defined as CHD death or non-fatal MI plus stroke, revascularization procedures, angina [in hospital or outpatient], heart failure [in hospital or outpatient], stroke, and peripheral arterial disease). Outcomes were reported by the clinical investigator and supported, when possible, by death certificates and hospitalization records. For a more detailed description of ALLHAT outcomes, see Davis et al. [28].

### Genotyping

Anonymized samples containing DNA were collected on FTA paper (Fitzco Inc., Maple Plain, MN) and processed as described by Arnett et al. [26]. Genotyping was performed using a multiplex PCR and a linear immobilized probe research assay for candidate markers (Roche Molecular Systems, Pleasanton, CA, USA) as described by Burns et al. [30]. GenHAT genotyped two SNPs in *MMP9* (R279Q, rs17576 and R668Q, rs2274756) and two SNPs in *MMP12* (N122S, rs652438 and −82A>G, rs2276109). The polymorphisms were genotyped in the context of a larger panel of variants selected by the assay's designer as candidates for cardiovascular disease. Genotyping QC was accomplished by preparing a set of 10 trays of replicate DNA samples (n = 767; 2% of GenHAT cohort) which were genotyped along with the others. For the four variants reported on here, correspondence between replicate genotype calls ranged from 97.6% to 99.1%.

### Statistical analysis

STATA version 9.2 (STATA Corporation, College Station, TX) was used for all analyses. Hardy-Weinberg equilibrium (HWE) tests were performed using chi-square tests. To test for baseline differences between treatment groups, we used ANOVA for continuous variables and chi-square tests for categorical variables. Cox proportional regression was used for testing the main effects of MMP genotypes on the outcomes as well as the genotype-by-treatment interactions (the pharmacogenetic effects), resulting in hazard ratios (HR) and ratios of hazard ratios point estimates, respectively. To test the main effects of the genotypes on the outcomes, we adjusted for age; gender; ethnicity (Hispanic status); race (5 self-reported categories: White, Black, American Indian/Alaskan native, Asian/Pacific Islander, and “other”); smoking status; diabetes status; aspirin use; and baseline values for body mass index (BMI), systolic and diastolic blood pressures, and HDL and LDL cholesterol. To test for genotype-by-treatment interactions, we created a genotype x treatment parameter and did three separate comparisons: chlorthalidone versus amlodipine, chlorthalidone versus lisinopril, and lisinopril versus amlodipine. We tested three genetic models for the pharmacogenetic effects: additive (3 genotype categories with a 2 degrees of freedom [df] interaction test), dominant (collapsing minor homozygote group with heterozygote group resulting in 2 genotype groups with a 1 df interaction test), and recessive (collapsing common homozygote group with heterozygote group resulting in 2 genotype groups with a 1 df interaction test).

The previously published GenHAT design paper outlined six primary, *a priori* hypotheses; however, these hypotheses did not include testing the pharmacogenetic effect of *MMP9* or *MMP12* variants. Therefore, secondary investigations such as this MMP study are considered exploratory and, as such, are not adjusted for multiple comparisons. Since we performed multiple statistical tests of the pharmacogenetic effects of *MMP9* and *MMP12* variants, caution must be exercised in pronouncing any findings reported here as statistically significant.

## Results

Table 1 shows the baseline characteristics of the study subjects (n = 38,698, participants with at least one variant included in these analyses). There were no significant differences between groups except for HDL cholesterol (*P* = 0.04), which was higher in those randomized to amlodipine versus chlorthalidone or lisinopril.

**Table 1:** Baseline characteristics for participants (n = 38,698) by treatment group, mean (SD) unless otherwise noted.

Characteristic	Chlorthalidone	Amlodipine	Lisinopril	Doxazosin	*P*-value*
Sample size, n (%) by treatment	13,928 (36.0)	8,243 (21.3)	8,273 (21.4)	8,254 (21.3)	
Age (y)	66.8 (7.7)	66.9 (7.7)	66.8 (7.8)	66.7 (7.7)	0.74
Race					
White, n (col %)	8,451 (60.7)	4,999 (60.7)	5,025 (60.7)	4,976 (60.3)	
Black, n (col %)	4,770 (34.3)	2,859 (34.7)	2,839 (34.3)	2,890 (35.0)	
American Indian/Alaskan native, n (col %)	26 (0.2)	19 (0.2)	18 (0.2)	10 (0.1)	
Asian/Pacific Islander, n (col %)	170 (1.2)	97 (1.2)	85 (1.0)	95 (1.2)	
Other, n (col %)	511 (3.7)	269 (3.3)	306 (3.7)	283 (3.4)	0.64
Hispanic, n (%)	2,722 (19.5)	1,558 (18.9)	1,639 (19.8)	1,618 (19.6)	0.86
Women, n (%)	6,552 (47.0)	3,926 (47.6)	3,831 (46.3)	3,817 (46.2)	0.22
Previous antihypertensive treatment, n (%)	12,571 (90.3)	7,472 (90.7)	7,454 (90.1)	7,447 (90.2)	0.66
Blood pressure at baseline, mm Hg					
All participants					
SBP	146.2 (15.7)	146.2 (15.7)	146.5 (15.6)	146.3 (15.8)	0.67
DBP	84.1 (10.0)	83.9 (10.2)	84.1 (10.0)	83.9 (10.0)	0.41
Treated at baseline					
SBP	145.2 (15.6)	145.1 (15.6)	145.4 (15.5)	145.2 (15.7)	0.75
DBP	83.5 (10.0)	83.3 (10.1)	83.6 (9.9)	83.4 (9.9)	0.30
Untreated at baseline					
SBP	156.1 (12.0)	156.6 (12.2)	156.4 (12.3)	156.8 (12.5)	0.57
DBP	89.5 (9.0)	89.7 (9.5)	89.1 (9.3)	89.4 (9.5)	0.63
Eligibility risk factors					
Current cigarette smoker, n (%)	3,061 (22.0)	1,810 (22.0)	1,817 (22.0)	1,795 (21.8)	0.98
Type 2 diabetes, n (%)	4,983 (35.8)	3,008 (36.5)	2,899 (35.0)	2,891 (35.0)	0.15
HDL cholesterol <35 mg/dL, n (%)	1,666 (12.0)	939 (11.4)	976 (11.8)	973 (11.8)	0.65
LVH by electrocardiogram, n (%)	2,255 (16.2)	1,398 (17.0)	1,342 (16.2)	1,354 (16.4)	0.47
Body mass index, kg/m^2^	29.7 (6.1)	29.8 (6.3)	29.8 (6.2)	29.7 (5.9)	0.32
Fasting glucose, mg/dL	123.4 (58.6)	123.1 (57.5)	122.4 (55.6)	121.9 (55.9)	0.35
LDL cholesterol, mg/dL	136.0 (37.3)	135.7 (37.3)	135.8 (36.4)	135.2 (36.3)	0.60
HDL cholesterol, mg/dL	46.8 (14.9)	47.2 (14.7)	46.6 (14.6)	46.6 (14.4)	**0.04**
Fasting triglycerides, mg/dL	176.7 (130.9)	176.6 (132.8)	175.5 (138.5)	174.1 (137.6)	0.53
Aspirin use, n (%)	4,986 (35.8)	3,002 (36.4)	3,021 (36.5)	3,026 (36.7)	0.54
*MMP9* R279Q (A>G) (rs17576)					
AA	5,955 (42.8)	3,422 (41.6)	3,596 (43.6)	3,550 (43.1)	
AG	6,257 (45.0)	3,783 (46.0)	3,633 (44.0)	3,675 (44.6)	
GG	1,691 (12.2)	1,026 (12.5)	1,024 (12.4)	1,013 (12.3)	0.21
*MMP9* R668Q (G>A) (rs2274756)					
GG	10,004 (72.0)	5,827 (70.8)	5,916 (71.7)	5,939 (72.1)	
GA	3,564 (25.7)	2,196 (26.7)	2,121 (25.7)	2,085 (25.3)	
AA	326 (2.4)	207 (2.5)	219 (2.7)	211 (2.6)	0.33
*MMP12* −82A>G (rs2276109)					
AA	11,648 (84.0)	6,899 (84.0)	6,903 (83.8)	6,926 (84.3)	
AG	2,083 (15.0)	1,242 (15.1)	1,269 (15.4)	1,216 (14.8)	
GG	137 (1.0)	68 (0.8)	68 (0.8)	78 (1.0)	0.74
*MMP12* N122S (A>G) (rs652438)					
AA	11,029 (80.6)	6,562 (80.8)	6,652 (81.5)	6,565 (80.8)	
AG	2,423 (17.7)	1,415 (17.4)	1,400 (17.2)	1,426 (17.6)	
GG	235 (1.7)	145 (1.8)	111 (1.4)	132 (1.6)	0.31

SBP = systolic blood pressure, DBP = diastolic blood pressure, LVH = left ventricular hypertrophy.

*test of differences between genotype groups: ANOVA for continuous variables, chi-square for categorical variables.

The *MMP9* R279Q and *MMP12* −82A>G genotype frequencies were in Hardy-Weinberg equilibrium (HWE) when tested within race/ethnicity groups. The *MMP9* R668Q genotype frequencies were in HWE equilibrium for the Black, American Indian/Alaskan native, Asian/Pacific islander, and “other” groups, but not for the White group (*P* = 0.04). After stratifying Whites by Hispanic status, the *MMP9* R668Q genotype frequencies were in HWE for non-Hispanic Whites, but not for Hispanic Whites (*P* = 0.001). The *MMP12* N122S genotype frequencies were in HWE for the Asian/Pacific islander and “other” groups, but not for the Black or White groups. The American Indian/Alaskan native group included no minor *MMP12* N122S homozygotes (ie, GG). After stratifying Whites and Blacks by Hispanic status, *MMP12* N122S genotype frequencies were in HWE for all groups except Black non-Hispanics (*P* = 0.0003).

### Main effects of *MMP9* and *MMP12* variants on clinical outcomes

The effects of ALLHAT treatment assignments on CVD outcomes have been published elsewhere [24], [25], [27]. Hazard ratios for main effects of genetic variants on ALLHAT clinical outcomes in GenHAT are shown in Table 2. After adjusting for baseline characteristics, the association between the *MMP9* R279Q variant and stroke was modestly significant, with participants carrying the minor G allele having decreased risk of stroke (HR = 1.00 for AA, 0.87 for GA, 0.91 for GG; *P* = 0.04, additive genetic model). The associations between the *MMP12* N122S variant and CHD and CCHD were also modestly significant (*P* = 0.04 and *P* = 0.01, respectively), with participants with the minor allele homozygote (GG) genotype having a 30% increased risk of CHD or CCHD compared to participants having the common AA genotype (for CHD HR = 1.00 for AA, 0.95 for AG, 1.36 for GG; *P* = 0.04; for CCHD HR = 1.00 for AA, 0.95 for AG, 1.31 for GG; *P* = 0.01). The effects of the *MMP9* R668Q and *MMP12* −82A>G variants were not significant for any outcome.

**Table 2:** Main effects of MMP genetic variants on clinical outcomes, all treatments combined.

	Number of events (event rate per 1000 p-y)						Adjusted* Hazard Ratios (95% CI)					
Genotype	CHD	CCHD	CCVD	ACM	Stroke	CHF	CHD	CCHD	CCVD	ACM	Stroke	CHF
*MMP9* R279Q (rs17576)												
AA	1373	2573	4368	2268	777	1149	1.00	1.00	1.00	1.00	1.00	1.00
	(19.0)	(37.3)	(68.1)	(29.0)	(10.6)	(15.9)						
GA	1478	2738	4529	2319	731	1158	1.00	1.01	0.97	0.96	0.87	0.92
	(19.5)	(37.8)	(67.2)	(28.1)	(9.5)	(15.3)	(0.93–1.08)	(0.96–1.07)	(0.93–1.02)	(0.90–1.02)	(0.79–0.97)	(0.85–1.00)
GG	420	767	1275	618	202	347	1.00	1.01	0.99	0.93	0.91	1.02
	(20.3)	(38.9)	(69.3)	(27.4)	(9.6)	(16.8)	(0.90–1.14)	(0.93–1.10)	(0.93–1.06)	(0.85–1.02)	(0.78–1.07)	(0.90–1.16)
*P*							0.99	0.92	0.51	0.20	**0.04**	0.10
*MMP9* R668Q (rs2274756)												
GG	2360	4376	7311	3738	1240	1897	1.00	1.00	1.00	1.00	1.00	1.00
	(19.6)	(38.0)	(68.3)	(28.5)	(10.2)	(15.7)						
GA	817	1538	2579	1323	425	688	0.95	0.97	0.97	0.97	0.94	0.99
	(18.6)	(36.6)	(66.1)	(27.7)	(9.5)	(15.7)	(0.87–1.03)	(0.92–1.04)	(0.93–1.02)	(0.91–1.03)	(0.84–1.06)	(0.90–1.09)
AA	94	164	275	133	45	67	1.15	1.10	1.06	0.96	0.99	1.00
	(22.4)	(41.0)	(74.0)	(29.3)	(10.5)	(16.0)	(0.93–1.43)	(0.93–1.29)	(0.94–1.21)	(0.80–1.15)	(0.72–1.35)	(0.77–1.29)
*P*							0.16	0.34	0.31	0.59	0.61	0.98
*MMP12* −82A>G (rs2276109)												
AA	2692	4991	8409	4409	1445	2161	1.00	1.00	1.00	1.00	1.00	1.00
	(19.1)	(37.0)	(67.0)	(28.7)	(10.1)	(15.3)						
AG	535	1008	1624	736	245	449	0.97	0.96	0.96	0.93	1.00	1.05
	(20.8)	(41.4)	(71.9)	(26.5)	(9.4)	(17.5)	(0.88–1.08)	(0.90–1.04)	(0.91–1.02)	(0.85–1.01)	(0.86–1.15)	(0.94–1.18)
GG	35	67	116	47	19	32	0.99	1.00	1.12	0.97	1.32	1.26
	(22.4)	(45.2)	(87.3)	(27.9)	(12.0)	(20.6)	(0.68–1.42)	(0.77–1.29)	(0.92–1.36)	(0.71–1.32)	(0.81–2.13)	(0.86–1.83)
*P*							0.87	0.63	0.24	0.20	0.53	0.36
*MMP12* N122S (rs652438)												
AA	2651	4969	8210	4143	1334	2126	1.00	1.00	1.00	1.00	1.00	1.00
	(19.7)	(38.7)	(68.8)	(28.4)	(9.8)	(15.8)						
AG	503	911	1621	893	321	432	0.95	0.95	0.96	0.98	1.10	0.97
	(17.2)	(32.4)	(61.9)	(28.2)	(10.9)	(14.9)	(0.86–1.05)	(0.88–1.02)	(0.91–1.02)	(0.91–1.06)	(0.96–1.25)	(0.87–1.09)
GG	60	102	169	82	29	45	1.36	1.31	1.14	0.95	1.01	1.13
	(22.5)	(40.1)	(71.2)	(27.3)	(10.7)	(16.7)	(1.04–1.79)	(1.06–1.62)	(0.96–1.34)	(0.76–1.20)	(0.68–1.49)	(0.83–1.55)
*P*							**0.04**	**0.01**	0.12	0.83	0.39	0.64

CHD, coronary heart disease defined as fatal CHD or nonfatal myocardial infarction; CCHD, combined CHD defined as CHD death or non-fatal myocardial infarction plus revascularization procedures plus hospitalized angina; CCVD, combined cardiovascular disease defined as CHD death or non-fatal MI plus stroke, revascularization procedures, angina, heart failure, stroke, and peripheral arterial disease; ACM, all-cause mortality; CHF, .chronic heart failure.

*adjusted for age, sex, race, Hispanic status, smoking status, diabetes status, aspirin use, and baseline values for BMI, SBP, DBP, HDL- and LDL-cholesterol.

### Pharmacogenetic effects of *MMP9* and *MMP12* variants on clinical outcomes

Tables 3, 4, 5, 6, 7, 8 show ALLHAT clinical outcome frequencies and rates by genotype and treatment group for CHD, CCHD, CCVD, ACM, stroke, and CHF, respectively. Also shown are genotype-specific treatment effects and gene-by-treatment interaction *P*-values for chlorthalidone versus amlodipine, chlorthalidone versus lisinopril, and lisinopril versus amlodipine. Treatment effects differed by genotype for several outcomes.

**Table 3:** Pharmacogenetic effects of MMP genetic variants on CHD.

	Number of events (Event rate per 1000 p-y)			Genotype-specific treatment effects (Hazard Ratio (95% CI))			Pharmacogenetic effects*		
Genotype	CHL	AML	LIS	C vs. A	C. vs. L	L vs. A	C vs. A	C. vs. L	L vs. A
*MMP9* R279Q (rs17576)									
AA	544	285	311	1.12	1.06	1.06	*P*_A_ = 0.11	*P*_A_ = 0.18	*P*_A_ = 0.56
	(20.0)	(17.9)	(18.9)	(0.97–1.29)	(0.92–1.21)	(0.90–1.24)			
AG	550	343	307	0.97	1.03	0.94	*P*_D_ = 0.07	*P*_D_ = 0.39	*P*_D_ = 0.38
	(19.0)	(19.7)	(18.5)	(0.84–1.11)	(0.89–1.18)	(0.81–1.10)			
GG	137	100	101	0.84	0.81	1.04	*P*_R_ = 0.13	*P*_R_ = 0.07	*P*_R_ = 0.78
	(17.7)	(21.1)	(22.0)	(0.65–1.08)	(0.62–1.04)	(0.79–1.37)			
*MMP9* R668Q (rs2274756)									
GG	902	523	517	1.01	1.02	0.98	*P*_A_ = 0.13	*P*_A_ = 0.45	*P*_A_ = 0.61
	(19.7)	(19.5)	(19.2)	(0.91–1.12)	(0.92–1.14)	(0.87–1.11)			
GA	303	177	180	1.07	0.99	1.08	*P*_D_ = 0.97	*P*_D_ = 0.53	*P*_D_ = 0.60
	(18.3)	(17.2)	(18.6)	(0.89–1.29)	(0.82–1.19)	(0.88–1.33)			
AA	25	27	24	0.59	0.74	0.84	*P*_R_ = 0.05	*P*_R_ = 0.22	*P*_R_ = 0.50
	(16.9)	(28.5)	(23.8)	(0.34–1.02)	(0.42–1.30)	(0.48–1.46)			
*MMP12* −82A>G (rs2276109)									
AA	1006	610	591	0.98	1.00	0.98	*P*_A_ = 0.52	*P*_A_ = 0.19	*P*_A_ = 0.13
	(18.8)	(19.2)	(18.8)	(0.88–1.08)	(0.90–1.10)	(0.88–1.10)			
AG	200	108	125	1.14	0.98	1.16	*P*_D_ = 0.25	*P*_D_ = 0.77	*P*_D_ = 0.44
	(20.9)	(18.4)	(21.3)	(0.90–1.44)	(0.79–1.23)	(0.89–1.50)			
GG	18	9	3	1.00	3.25	0.33	*P*_R_ = 0.96	*P*_R_ = 0.07	*P*_R_ = 0.10
	(28.9)	(28.3)	(9.3)	(0.45–2.22)	(0.96–11.0)	(0.09–1.22)			
*MMP12* N122S (rs652438)									
AA	1003	593	563	1.01	1.07	0.94	*P*_A_ = 0.91	***P*** **_A_ = 0.005**	***P*** **_A_ = 0.041**
	(19.8)	(19.6)	(18.5)	(0.91–1.12)	(0.97–1.19)	(0.84–1.06)			
AG	182	113	128	0.96	0.80	1.19	*P*_D_ = 0.66	***P*** **_D_ = 0.005**	***P*** **_D_ = 0.037**
	(16.3)	(17.0)	(20.3)	(0.76–1.21)	(0.64–1.01)	(0.93–1.54)			
GG	21	13	19	0.97	0.49	1.93	*P*_R_ = 0.89	***P*** **_R_ = 0.019**	
	(19.7)	(20.6)	(40.9)	(0.48–1.93)	(0.26–0.91)	(0.95–3.92)			

*P*_A_ = *P*-value for additive genetic model, *P*_D_ = *P*-value for dominant genetic model, *P*_R_ = *P*-value for recessive genetic model.

*P-value for gene-by-treatment interaction.

CHD, coronary heart disease defined as fatal CHD or nonfatal myocardial infarction; CHL, chlorthalidone treatment group; AML, amlodipine treatment group; LIS, lisinopril treatment group.

**Table 4:** Pharmacogenetic effects of MMP genetic variants on CCHD.

	Number of events (event rate per 1000 p-y)			Genotype-specific treatment effects: Hazard Ratio (95% CI)			Pharmacogenetic effects*		
Genotype	CHL	AML	LIS	C vs. A	C. vs. L	L vs. A	C vs. A	C. vs. L	L vs. A
*MMP9* R279Q (rs17576)									
AA	952	556	569	1.00	1.01	0.99	*P*_A_ = 0.22	*P*_A_ = 0.12	*P*_A_ = 0.71
	(36.5)	(36.6)	(36.3)	(0.90–1.11)	(0.91–1.12)	(0.88–1.11)			
AG	1022	602	599	1.03	0.97	1.06	*P*_D_ = 0.91	*P*_D_ = 0.29	*P*_D_ = 0.40
	(37.2)	(36.1)	(38.1)	(0.93–1.14)	(0.88–1.08)	(0.94–1.18)			
GG	256	182	186	0.85	0.81	1.06	*P*_R_ = 0.09	***P*** **_R_ = 0.045**	*P*_R_ = 0.78
	(34.5)	(40.6)	(42.9)	(0.70–1.03)	(0.67–0.97)	(0.86–1.30)			
*MMP9* R668Q (rs2274756)									
GG	1615	946	970	1.00	0.97	1.03	*P*_A_ = 0.12	*P*_A_ = 0.30	*P*_A_ = 0.84
	(36.9)	(36.9)	(37.9)	(0.92–1.08)	(0.90–1.05)	(0.94–1.12)			
GA	565	350	346	1.01	0.96	1.05	*P*_D_ = 0.65	*P*_D_ = 0.54	*P*_D_ = 0.89
	(35.9)	(35.7)	(37.6)	(0.88–1.15)	(0.84–1.09)	(0.91–1.22)			
AA	45	44	43	0.64	0.71	0.93	***P*** **_R_ = 0.038**	*P*_R_ = 0.13	*P*_R_ = 0.60
	(31.6)	(49.2)	(45.4)	(0.42–0.97)	(0.47–1.08)	(0.61–1.42)			
*MMP12* −82A>G (rs2276109)									
AA	1817	1109	1120	0.97	0.95	1.03	*P*_A_ = 0.48	*P*_A_ = 0.18	*P*_A_ = 0.76
	(35.5)	(36.5)	(37.6)	(0.90–1.05)	(0.88–1.02)	(0.95–1.12)			
AG	370	218	226	1.04	1.00	1.04	*P*_D_ = 0.35	*P*_D_ = 0.31	*P*_D_ = 0.95
	(40.8)	(39.3)	(40.8)	(0.88–1.23)	(0.85–1.18)	(0.86–1.25)			
GG	32	12	9	1.35	1.91	0.72	*P*_R_ = 0.33	*P*_R_ = 0.08	*P*_R_ = 0.46
	(54.1)	(39.4)	(29.1)	(0.70–2.63)	(0.91–4.01)	(0.30–1.71)			
*MMP12* N122S (rs652438)									
AA	1831	1092	1092	1.00	1.00	1.00	*P*_A_ = 0.45	***P*** **_A_ = 0.023**	*P*_A_ = 0.14
	(38.0)	(37.8)	(37.8)	(0.93–1.08)	(0.93–1.08)	(0.92–1.09)			
AG	319	209	227	0.90	0.79	1.15	*P*_D_ = 0.39	***P*** **_D_ = 0.008**	*P*_D_ = 0.10
	(29.7)	(32.9)	(37.7)	(0.76–1.07)	(0.66–0.93)	(0.95–1.38)			
GG	40	20	24	1.18	0.74	1.58	*P*_R_ = 0.52	*P*_R_ = 0.29	*P*_R_ = 0.14
	(39.1)	(33.1)	(53.5)	(0.69–2.01)	(0.45–1.23)	(0.87–2.85)			

*P*_A_ = *P*-value for additive genetic model, *P*_D_ = *P*-value for dominant genetic model, *P*_R_ = *P*-value for recessive genetic model.

*P-value for gene-by-treatment interaction.

CCHD, combined CHD defined as CHD death or non-fatal myocardial infarction plus revascularization procedures plus hospitalized angina; CHL, chlorthalidone treatment group; AML, amlodipine treatment group; LIS, lisinopril treatment group.

**Table 5:** Pharmacogenetic effects of MMP genetic variants on CCVD.

	Number of events (event rate per 1000 p-y)			Genotype-specific treatment effects: Hazard Ratio (95% CI)			Pharmacogenetic effects*		
Genotype	CHL	AML	LIS	C vs. A	C. vs. L	L vs. A	C vs. A	C. vs. L	L vs. A
*MMP9* R279Q (rs17576)									
AA	1552	952	963	0.94	0.96	0.98	***P*** **_A_ = 0.038**	***P*** **_A_ = 0.05**	*P*_A_ = 0.15
	(63.5)	(67.6)	(66.2)	(0.87–1.02)	(0.89–1.04)	(0.90–1.07)			
AG	1631	978	1012	1.01	0.91	1.11	*P*_D_ = 0.67	*P*_D_ = 0.10	*P*_D_ = 0.06
	(63.4)	(62.9)	(70.0)	(0.93–1.09)	(0.84–0.98)	(1.02–1.21)			
GG	413	301	303	0.81	0.78	1.04	***P*** **_R_ = 0.024**	***P*** **_R_ = 0.026**	*P*_R_ = 0.98
	(58.8)	(72.5)	(75.7)	(0.70–0.94)	(0.67–0.90)	(0.89–1.22)			
*MMP9* R668Q (rs2274756)									
GG	2602	1600	1617	0.94	0.93	1.01	*P*_A_ = 0.36	*P*_A_ = 0.33	*P*_A_ = 0.16
	(63.4)	(67.3)	(68.1)	(0.89–1.00)	(0.88–0.99)	(0.94–1.08)			
GA	907	562	597	1.00	0.87	1.15	*P*_D_ = 0.52	*P*_D_ = 0.17	*P*_D_ = 0.07
	(61.4)	(61.3)	(70.9)	(0.90–1.11)	(0.78–0.96)	(1.03–1.29)			
AA	82	65	69	0.80	0.78	1.02	*P*_R_ = 0.28	*P*_R_ = 0.35	*P*_R_ = 0.88
	(61.9)	(77.4)	(78.9)	(0.58–1.11)	(0.56–1.07)	(0.73–1.44)			
*MMP12* −82A>G (rs2276109)									
AA	2942	1857	1890	0.93	0.90	1.04	*P*_A_ = 0.21	*P*_A_ = 0.06	*P*_A_ = 0.35
	(61.3)	(65.7)	(68.6)	(0.88–0.99)	(0.84–0.95)	(0.98–1.11)			
AG	588	343	371	1.05	0.95	1.10	*P*_D_ = 0.08	*P*_D_ = 0.16	*P*_D_ = 0.74
	(69.4)	(66.2)	(72.8)	(0.92–1.20)	(0.84–1.09)	(0.95–1.27)			
GG	53	23	17	1.16	1.75	0.70	*P*_R_ = 0.41	***P*** **_R_ = 0.025**	*P*_R_ = 0.19
	(100.8)	(85.9)	(59.0)	(0.71–1.90)	(1.01–3.02)	(0.37–1.31)			
*MMP12* N122S (rs652438)									
AA	2903	1800	1822	0.96	0.94	1.02	*P*_A_ = 0.52	*P*_A_ = 0.06	*P*_A_ = 0.19
	(64.2)	(67.1)	(68.3)	(0.90–1.01)	(0.89–1.00)	(0.95–1.09)			
AG	566	350	388	0.96	0.81	1.18	*P*_D_ = 0.76	***P*** **_D_ = 0.024**	*P*_D_ = 0.08
	(56.3)	(58.8)	(69.5)	(0.84–1.09)	(0.71–0.92)	(1.02–1.36)			
GG	59	44	37	0.76	0.70	1.10	*P*_R_ = 0.25	*P*_R_ = 0.19	*P*_R_ = 0.83
	(60.9)	(80.2)	(88.6)	(0.52–1.13)	(0.46–1.05)	(0.71–1.71)			

*P*_A_ = *P*-value for additive genetic model, *P*_D_ = *P*-value for dominant genetic model, *P*_R_ = *P*-value for recessive genetic model.

*P-value for gene-by-treatment interaction.

CCVD, combined cardiovascular disease defined as CHD death or non-fatal MI plus stroke, revascularization procedures, angina, heart failure, stroke, and peripheral arterial disease; CHL, chlorthalidone treatment group; AML, amlodipine treatment group; LIS, lisinopril treatment group.

**Table 6:** Pharmacogenetic effects of MMP genetic variants on ACM.

	Number of events (event rate per 1000 p-y)			Genotype-specific treatment effects: Hazard Ratio (95% CI)			Pharmacogenetic effects*		
Genotype	CHL	AML	LIS	C vs. A	C. vs. L	L vs. A	C vs. A	C. vs. L	L vs. A
*MMP9* R279Q (rs17576)									
AA	858	465	519	1.08	1.00	1.07	*P*_A_ = 0.72	*P*_A_ = 0.87	*P*_A_ = 0.76
	(29.6)	(27.5)	(29.5)	(0.96–1.21)	(0.90–1.12)	(0.95–1.22)			
AG	883	510	526	1.04	0.97	1.08	*P*_D_ = 0.53	*P*_D_ = 0.67	*P*_D_ = 0.85
	(28.6)	(27.5)	(29.6)	(0.93–1.16)	(0.87–1.08)	(0.96–1.22)			
GG	229	141	138	0.98	1.00	0.98	*P*_R_ = 0.50	*P*_R_ = 0.87	*P*_R_ = 0.46
	(27.6)	(28.0)	(27.6)	(0.79–1.21)	(0.81–1.24)	(0.77–1.24)			
*MMP9* R668Q (rs2274756)									
GG	1420	790	859	1.05	0.97	1.08	*P*_A_ = 0.97	*P*_A_ = 0.83	*P*_A_ = 0.81
	(29.1)	(27.6)	(29.8)	(0.96–1.15)	(0.89–1.06)	(0.98–1.19)			
GA	501	294	291	1.05	1.02	1.03	*P*_D_ = 0.94	*P*_D_ = 0.54	*P*_D_ = 0.55
	(28.4)	(27.0)	(27.8)	(0.91–1.21)	(0.89–1.18)	(0.87–1.21)			
AA	47	30	32	1.00	1.06	0.96	*P*_R_ = 0.81	*P*_R_ = 0.87	*P*_R_ = 0.71
	(30.0)	(30.1)	(29.7)	(0.63–1.57)	(0.67–1.66)	(0.58–1.58)			
*MMP12* −82A>G (rs2276109)									
AA	1646	951	1008	1.03	0.96	1.07	*P*_A_ = 0.17	*P*_A_ = 0.19	*P*_A_ = 0.12
	(28.9)	(28.1)	(29.9)	(0.95–1.11)	(0.89–1.04)	(0.98–1.16)			
AG	297	148	168	1.22	1.09	1.12	*P*_D_ = 0.19	*P*_D_ = 0.15	*P*_D_ = 0.94
	(29.1)	(24.0)	(26.9)	(1.00–1.49)	(0.90–1.32)	(0.90–1.40)			
GG	21	14	5	0.74	2.04	0.37	*P*_R_ = 0.31	*P*_R_ = 0.16	***P*** **_R_ = 0.043**
	(30.8)	(42.1)	(15.1)	(0.37–1.45)	(0.77–5.42)	(0.13–1.04)			
*MMP12* N122S (rs652438)									
AA	1540	909	926	1.01	1.00	1.01	***P*** **_A_ = 0.021**	*P*_A_ = 0.23	***P*** **_A_ = 0.013**
	(28.5)	(28.3)	(28.5)	(0.93–1.10)	(0.93–1.09)	(0.92–1.10)			
AG	365	164	220	1.32	0.95	1.39	***P*** **_D_ = 0.022**	*P*_D_ = 0.33	***P*** **_D_ = 0.004**
	(30.7)	(23.3)	(32.3)	(1.10–1.59)	(0.80–1.12)	(1.14–1.71)			
GG	29	22	21	0.81	0.63	1.28	*P*_R_ = 0.35	*P*_R_ = 0.11	*P*_R_ = 0.52
	(24.8)	(30.9)	(39.6)	(0.47–1.42)	(0.36–1.10)	(0.70–2.32)			

*P*_A_ = *P*-value for additive genetic model, *P*_D_ = *P*-value for dominant genetic model, *P*_R_ = *P*-value for recessive genetic model.

*P-value for gene-by-treatment interaction.

ACM, all-cause mortality; CHL, chlorthalidone treatment group; AML, amlodipine treatment group; LIS, lisinopril treatment group.

**Table 7:** Pharmacogenetic effects of MMP genetic variants on stroke.

	Number of events (event rate per 1000 p-y)			Genotype-specific treatment effects: Hazard Ratio (95% CI)			Pharmacogenetic effects*		
Genotype	CHL	AML	LIS	C vs. A	C. vs. L	L vs. A	C vs. A	C. vs. L	L vs. A
*MMP9* R279Q (rs17576)									
AA	288	146	180	1.15	0.96	1.20	*P*_A_ = 0.63	*P*_A_ = 0.29	*P*_A_ = 0.47
	(10.4)	(9.0)	(10.8)	(0.94–1.40)	(0.80–1.16)	(0.96–1.49)			
AG	253	145	185	1.05	0.78	1.35	*P*_D_ = 0.41	*P*_D_ = 0.18	*P*_D_ = 0.68
	(8.6)	(8.2)	(11.1)	(0.86–1.29)	(0.65–0.94)	(1.08–1.67)			
GG	70	45	45	0.95	0.93	1.02	*P*_R_ = 0.46	*P*_R_ = 0.75	*P*_R_ = 0.34
	(8.9)	(9.4)	(9.6)	(0.65–1.38)	(0.64–1.35)	(0.68–1.55)			
*MMP9* R668Q (rs2274756)									
GG	456	247	286	1.08	0.93	1.16	*P*_A_ = 0.12	*P*_A_ = 0.16	*P*_A_ = 0.15
	(9.8)	(9.1)	(10.5)	(0.93–1.26)	(0.81–1.08)	(0.98–1.37)			
GA	139	87	114	0.99	0.71	1.39	*P*_D_ = 0.99	*P*_D_ = 0.10	*P*_D_ = 0.15
	(8.3)	(8.4)	(11.6)	(0.76–1.30)	(0.56–0.92)	(1.05–1.84)			
AA	17	3	11	3.65	1.04	3.50	***P*** **_R_ = 0.048**	*P*_R_ = 0.62	*P*_R_ = 0.11
	(11.3)	(3.1)	(10.7)	(1.07–12.5)	(0.49–2.23)	(0.98–12.6)			
*MMP12* −82A>G (rs2276109)									
AA	507	283	356	1.06	0.83	1.28	*P*_A_ = 0.42	*P*_A_ = 0.18	*P*_A_ = 0.29
	(9.3)	(8.8)	(11.2)	(0.92–1.23)	(0.73–0.95)	(1.10–1.50)			
AG	98	48	53	1.25	1.14	1.09	*P*_D_ = 0.57	*P*_D_ = 0.07	*P*_D_ = 0.29
	(10.1)	(8.1)	(8.9)	(0.88–1.76)	(0.82–1.60)	(0.74–1.62)			
GG	6	5	2	0.59	1.58	0.43	*P*_R_ = 0.31	*P*_R_ = 0.50	*P*_R_ = 0.16
	(9.2)	(15.8)	(6.1)	(0.18–1.94)	(0.32–7.81)	(0.08–2.25)			
*MMP12* N122S (rs652438)									
AA	483	261	315	1.10	0.92	1.20	*P*_A_ = 0.62	*P*_A_ = 0.40	*P*_A_ = 0.43
	(9.4)	(8.5)	(10.2)	(0.95–1.28)	(0.80–1.06)	(1.02–1.42)			
AG	108	62	83	1.04	0.74	1.41	*P*_D_ = 0.56	*P*_D_ = 0.22	*P*_D_ = 0.60
	(9.7)	(9.3)	(13.1)	(0.76–1.42)	(0.56–0.99)	(1.01–1.96)			
GG	11	9	5	0.72	1.01	0.71	*P*_R_ = 0.36	*P*_R_ = 0.80	*P*_R_ = 0.32
	(10.2)	(14.2)	(10.1)	(0.30–1.74)	(0.35–2.91)	(0.24–2.11)			

*P*_A_ = *P*-value for additive genetic model, *P*_D_ = *P*-value for dominant genetic model, *P*_R_ = *P*-value for recessive genetic model.

*P-value for gene-by-treatment interaction.

CHL, chlorthalidone treatment group; AML, amlodipine treatment group; LIS, lisinopril treatment group.

**Table 8:** Pharmacogenetic effects of MMP genetic variants on CHF.

	Number of events (event rate per 1000 p-y)			Genotype-specific treatment effects: Hazard Ratio (95% CI)			Pharmacogenetic effects*		
Genotype	CHL	AML	LIS	C vs. A	C. vs. L	L vs. A	C vs. A	C. vs. L	L vs. A
*MMP9* R279Q (rs17576)									
AA	359	283	239	0.73	0.90	0.82	*P*_A_ = 0.53	*P*_A_ = 0.41	*P*_A_ = 0.29
	(13.1)	(17.8)	(14.5)	(0.63–0.86)	(0.76–1.06)	(0.69–0.97)			
AG	346	281	255	0.73	0.77	0.95	*P*_D_ = 0.68	*P*_D_ = 0.20	*P*_D_ = 0.38
	(11.9)	(16.2)	(15.5)	(0.63–0.86)	(0.65–0.90)	(0.80–1.13)			
GG	97	95	70	0.61	0.82	0.75	*P*_R_ = 0.26	*P*_R_ = 0.92	*P*_R_ = 0.35
	(12.4)	(20.2)	(15.2)	(0.46–0.81)	(0.60–1.11)	(0.55–1.03)			
*MMP9* R668Q (rs2274756)									
GG	591	463	403	0.74	0.85	0.87	*P*_A_ = 0.57	*P*_A_ = 0.59	*P*_A_ = 0.76
	(12.8)	(17.3)	(15.0)	(0.65–0.83)	(0.75–0.97)	(0.76–0.99)			
GA	192	175	149	0.67	0.75	0.90	*P*_D_ = 0.34	*P*_D_ = 0.33	*P*_D_ = 0.93
	(11.5)	(17.2)	(15.4)	(0.55–0.82)	(0.60–0.93)	(0.72–1.12)			
AA	17	19	14	0.57	0.83	0.69	*P*_R_ = 0.49	*P*_R_ = 0.97	*P*_R_ = 0.49
	(11.5)	(20.1)	(13.8)	(0.30–1.10)	(0.41–1.68)	(0.35–1.37)			
*MMP12* −82A>G (rs2276109)									
AA	628	546	467	0.67	0.78	0.86	*P*_A_ = 0.05	***P*** **_A_ = 0.040**	*P*_A_ = 0.15
	(11.6)	(17.3)	(14.9)	(0.60–0.76)	(0.69–0.88)	(0.76–0.98)			
AG	154	99	96	0.95	0.98	0.97	***P*** **_D_ = 0.015**	***P*** **_D_ = 0.044**	*P*_D_ = 0.77
	(16.0)	(17.0)	(16.4)	(0.74–1.22)	(0.76–1.26)	(0.73–1.28)			
GG	14	9		0.77	3.76	0.21	*P*_R_ = 0.83	***P*** **_R_ = 0.048**	*P*_R_ = 0.07
	(22.4)	(28.8)	(6.1)	(0.33–1.78)	(0.85–16.6)	(0.04–0.95)			
*MMP12* N122S (rs652438)									
AA	639	522	450	0.72	0.84	0.86	*P*_A_ = 0.98	*P*_A_ = 0.41	*P*_A_ = 0.55
	(12.5)	(17.3)	(14.8)	(0.64–0.81)	(0.75–0.95)	(0.76–0.97)			
AG	131	108	96	0.72	0.77	0.93	*P*_D_ = 0.90	*P*_D_ = 0.37	*P*_D_ = 0.46
	(11.7)	(16.4)	(15.2)	(0.55–0.92)	(0.59–1.00)	(0.70–1.22)			
GG	14	12	12	0.68	0.52	1.31	*P*_R_ = 0.84	*P*_R_ = 0.24	*P*_R_ = 0.34
	(12.9)	(19.2)	(24.8)	(0.31–1.46)	(0.24–1.13)	(0.59–2.91)			

*P*_A_ = *P*-value for additive genetic model, *P*_D_ = *P*-value for dominant genetic model, *P*_R_ = *P*-value for recessive genetic model.

*P-value for gene-by-treatment interaction.

CHF; chronic heart failure; CHL, chlorthalidone treatment group; AML, amlodipine treatment group; LIS, lisinopril treatment group.

#### CHD, combined CHD, and combined CVD

Participants having the *MMP9* R668Q minor allele homozygote (AA) genotype had lower risk of CCHD when randomized to chlorthalidone versus amlodipine (HR = 0.64). No significant differences in CHD or CCHD were observed across AG or GG genotype-treatment groups. The *P*-value for the pharmacogenetic effect for CCHD in the recessive genetic model was 0.038.

Subjects with the *MMP9* R279Q GG genotype had lower risk of CCVD when randomized to chlorthalidone versus amlodipine or lisinopril (HR = 0.81 and 0.78, respectively) compared with participants with at least one copy of the A allele (pharmacogenetic effect *P* = 0.024 and *P* = 0.026 for chlorthalidone versus amlodipine and chlorthalidone versus lisinopril, respectively in recessive models). A similar effect was observed for the CCHD outcome, with only the chlorthalidone versus lisinopril comparison reaching a *P*-value<0.05 (*P* = 0.045 for the recessive model).

Participants having the *MMP12* N122S variant GG genotype had significantly increased rates (40.9 per 1000 p-y) of CHD if taking lisinopril versus any other genotype-treatment combination (16.3–20.6 per 1000 p-y). This difference led to a detectable pharmacogenetic effect for both chlorthalidone versus lisinopril and lisinopril versus amlodipine comparisons (*P*-values for pharmacogenetic effect ranged from 0.005 to 0.041 depending on genetic model). Results for CCHD and CCVD were consistent with this observation.

For the *MMP12* −82A>G variant, there was evidence of a pharmacogenetic effect on CCVD for the chlorthalidone versus lisinopril comparison. Homozygotes for the minor G allele had increased risk of CCVD when randomized to chlorthalidone versus lisinopril (HR = 1.75), while the common A allele carriers had slightly reduced risk (HR = 0.90 for AA group, HR = 0.95 for AG group; pharmacogenetic effect *P* = 0.025).

#### ACM

Participants with the *MMP12* N122S AG and GG genotypes experienced substantially increased ACM (39% and 28%, respectively) on lisinopril compared to those on amlodipine, while no differences in ACM were found with the common AA genotype-treatment combinations. The *P*-value for the pharmacogenetic effect was 0.013 when modeled additively and 0.004 when modeled dominantly. For the chlorthalidone versus amlodipine comparison, GG participants had reduced risk on chlorthalidone (HR = 0.81), while AG heterozygotes had increased risk (HR = 1.32). There was no treatment effect among the common AA homozygotes (HR = 1.01). The *P*-value for this pharmacogenetic effect was 0.021 when modeled additively.

Subjects having the *MMP12* −82A>G GG genotype had significantly lower ACM on lisinopril versus amlodipine (HR = 0.37), while those with at least one copy of the A allele had somewhat higher risk on lisinopril versus amlodipine (HR = 1.07 and 1.12 for AA and AG groups, respectively; *P* = 0.043 for the pharmacogenetic effect when modeled recessively).

#### Stroke

Those subjects having *MMP9* R668Q common GG genotype randomized to chlorthalidone had a slightly increased risk of stroke over those with the same genotype randomized to amlodipine (HR = 1.08), whereas participants with the AA genotype had a 3.65-fold increased risk of stroke when randomized to chlorthalidone versus amlodipine. There was no treatment effect among heterozygotes (HR = 0.99). The *P*-value for the pharmacogenetic effect was marginally significant (*P* = 0.048).

#### HF

A pharmacogenetic effect is suggested for the *MMP12* −82A>G polymorphism with both the chlorthalidone versus amlodipine group and the chlorthalidone versus lisinopril group comparisons. All subjects with data for the *MMP12* −82A>G polymorphism, regardless of genotype, showed decreased risk of HF on chlorthalidone versus amlodipine. However, there was a stronger protective effect of chlorthalidone versus amlodipine among those with the common AA genotype compared to G allele carriers (HR = 0.67 for AA group, HR = 0.95 for AG group, HR = 0.77 for GG group; *P* = 0.015 for pharmacogenetic effect when modeled dominantly). Among those with the GG genotype, a 3.76-fold increased risk of HF was found for those subjects randomized to chlorthalidone vs. lisinopril, whereas for those with the common AA genotype the risk of HF was reduced in the chlorthalidone group versus the lisinopril group (HR = 0.78). There was no difference in treatment effect among AG heterozygotes (HR = 0.98). Although this pharmacogenetic effect is significant (*P* = 0.040 for the additive model, *P* = 0.044 for the dominant model, *P* = 0.048 for the recessive model), it should be noted that there were few events (n = 2) in the GG genotype-lisinopril group.

### Implications of Hardy-Weinberg disequilibrium

Because *MMP12* N122S and *MMP9* R668Q variants were not in HWE for all ethnic subgroups, we tested whether the suggestive pharmacogenetic effects would be similar if we omitted the subgroups in HW disequilibrium. Therefore, for the *MMP12* N122S analysis we omitted the Black-non-Hispanic participants (31% of the overall population), and for the *MMP9* R668Q analysis we omitted the White-Hispanic participants (13% of the overall population). The results of these analyses showed that for all the findings presented in Tables 3, 4, 5, 6, 7, 8 for these two variants with a *P*-value less than 0.05, the pharmacogenetic effect (ratio of hazard ratios) was in the same direction and, for the most part, of similar magnitude (data not shown). Due in part to reduced sample sizes, the *P*-values varied in most cases. In some cases the association was slightly strengthened. In cases where association was attenuated, the most disparate finding when omitting the subgroup in HW disequilibrium was found for the pharmacogenetic effect of the *MMP9* R668Q variant on combined CHD when comparing chlorthalidone to amlodipine: for the full group modeled recessively, the ratio of hazard ratios was 0.64 (*P* = 0.04), whereas it was 0.71 (*P* = 0.13) when omitting the White-Hispanic group.

## Discussion

In this study we evaluated the pharmacogenetic effects of *MMP9* and *MMP12* variants on CHD, stroke, HF, combined CHD and CVD, and ACM. Our data provide evidence of pharmacogenetic associations between variants in the *MMP9* and *MMP12* genes and treatment for a variety of these cardiovascular outcomes: *MMP9* R668Q variant for both combined CHD and stroke; *MMP9* R279Q variant for the combined CVD outcome; and *MMP12* N122S variant for CHD, combined CHD, combined CVD, and ACM. Specifically, we found that patients having the *MMP9* R668Q AA genotype who were treated with chlorthalidone have a decreased risk of combined CHD but an increased risk of stroke compared to patients treated with amlodipine. Patients with the *MMP9* R279Q GG genotype treated with chlorthalidone had lower risk of combined CVD than patients treated with amlodipine or lisinopril. For those having either *MMP12* N122S AG or GG genotype, lisinopril treatment increased the risk of CHD, combined CHD, and combined CVD when compared to patients treated with chlorthalidone or a higher risk of CHD and ACM when compared to patients treated with amlodipine. In aggregate, these results indicate these variants may be useful in selecting appropriate antihypertensive agents to reduce risk of CVD.

Because some *MMP12* and *MMP9* variants were not in HWE for all ethnic subgroups, we investigated whether the pharmacogenetic effects would be similar if we omitted the subgroups in HW disequilibrium. These analyses showed that the pharmacogenetic effect was in the same direction and of similar magnitude with the reduced sample. We note that both the Black and Hispanic groups likely had diverse ancestry given that participants were recruited in the US, Canada, and the Caribbean; therefore, some degree of population substructure is expected. However, it should be emphasized that even if population substructure in the data leads to HW disequilibrium, which can lead to an increase in false-positive findings for *main effects* of genetic variants on outcomes, this should not explain any *pharmacogenetic findings* because participants were randomized to treatment; therefore, confounding from population substructure should be controlled because randomization in the context of a large sample size results in the same degree of population substructure in *each* treatment group. We conclude that, although unmeasured population substructure may be present, it is unlikely that the HW disequilibrium in some ethnic subgroups is driving our suggestive results.

MMP-9 may have a direct effect on plaque destabilization and may also serve as a biomarker predictive of mortality in patients with CVD [16]. As previously mentioned, elevated MMP-9 levels have been observed following acute coronary events [5]_ENREF_16. However, Johnson et al. suggested MMP-9 is activated during healing processes as opposed to causing rupture of plaques [31]. Based on our findings, chlorthalidone and amlodipine could potentially influence MMP-9 function. Chlorthalidone, a thiazide-like diuretic, acts on the Na^+^Cl^−^ symporter in the kidney, leading to greatly increased sodium and chloride excretion [32], [33]. Chlorthalidone's antihypertensive mechanism is not fully understood, and we are not aware of studies evaluating independent effects of chlorthalidone on MMP activity. However, Seeland et al. examined the effects of diuretic therapy in combination with an ACE inhibitor and found supplemental treatment with the diuretic furosemide did not provide additional benefit on extracellular matrix remodeling compared to ACE inhibitor alone [34]. Amlodipine is a CCB of the dihydropyridine class. It inhibits vascular smooth muscle contractions, causing increased blood flow to the heart and decreased peripheral arterial resistance and blood pressure [35]. Zervoudaki et al. reported that amlodipine increases plasma MMP-9 levels in hypertensive patients [8], [9]. However, a study involving lercanidipine (another dihydropyridine CCB) found decreased MMP-9 activity in hypertensives and suggested a mechanism involving the antioxidant effects of lercanidipine, which are shared by amlodipine [10]. A possible explanation for this discrepancy is that the amlodipine study used ELISA to measure MMP-9 level, whereas the more recent lercanidipine study used gel zymography [10]. Like other ACE inhibitors, lisinopril blocks the production of angiotensin II (a vasoconstrictor), thereby decreasing blood pressure. Like the MMPs, ACE is a zinc-dependent endopeptidase [11]. Studies have shown that ACE inhibition may also inhibit MMP levels [11], [12], [13], [14], [36], [37], [38]. Yamamoto et al. identified two potential interaction mechanisms between lisinopril and the active site of MMP-9 [38]. Lisinopril was shown to be stabilized in the active site by specific hydrogen bonds, and its hydrophobic group interacted preferentially with the S1 site compared to the S1′ site [38]. In their subsequent work, Yamamoto et al. and Takai et al. also found that different ACE inhibitors show differential binding affinities for MMP-9 [13], [36], [37], [38]. Sakata et al. observed that ACE inhibition directly lowers MMP activity in rats, thus preventing left ventricular remodeling [39]. The *MMP9* R279Q polymorphism is a glutamine to arginine substitution located in the catalytic domain of MMP-9 [16], [20] and may affect substrate binding [16]. The *MMP9* R668Q polymorphism is in the hemopexin-like domain and probably also functions in substrate binding, since removal of this domain disables the cleavage of triple-helix collagen [19].

MMP-12 is expressed in macrophages and epithelial cells and has been implicated in the progression of atherosclerosis, wound repair, and certain cancers [40], [41]. Like MMP-9, MMP-12 activity is increased following vascular injury [42]. Plasma MMP-12 levels are increased in patients with coronary artery disease and may, therefore, be an independent risk factor for coronary artery disease [43]. Morgan et al. observed higher MMP-12 levels in thin-cap atherosclerotic plaques (those considered more prone to rupture) versus thick-cap plaques. MMP-12 levels were also elevated in already ruptured plaques, suggesting that MMP-12 has a role in plaque stability [44]. Based on our findings, chlorthalidone, lisinopril, and amlodipine could potentially affect MMP-12 activity. To our knowledge, studies have not evaluated the effects of diuretics, ACE inhibitors, or CCBs on MMP-12. However, based on the finding that lercanidipine may inhibit macrophage function and the fact that macrophages secrete MMP-12, we hypothesize that CCBs may decrease MMP-12 levels [40], [45]. The MMP12 −82A>G polymorphism lies in the *MMP12* gene promoter [15]. The *MMP12* −82A>G polymorphism affects AP-1 binding affinity, with the A allele showing a higher affinity for the protein and, therefore, higher *MMP12* promoter activity [15], [18]. *MMP12* N122S is an asparagine to serine substitution located in the coding region of the *MMP12* hemopexin domain, which is responsible for gene activity [17], [18]. To our knowledge, no pharmacogenetic studies have been done on this combination of polymorphisms, drugs, and outcomes.

Our data suggest associations between the *MMP9* R279Q polymorphism and stroke and between the *MMP12* N122S polymorphism and combined CHD. The *MMP9* R279Q polymorphism has been associated with increased intima-media thickness [46], an independent predictor of MI and stroke risk [47]. Blankenberg et al. found that *MMP9* R279Q had no effect on plasma MMP-9 concentration but was associated with future cardiovascular events in patients with stable angina [16]. This polymorphism also influences aortic stiffness, which is a determinant of cardiovascular risk [48]. We also found a pharmacogenetic effect of the *MMP9* R668Q polymorphism to increased stroke risk, although this association may be due to the small number of events (17 in the chlorthalidone group, 3 in the amlodipine group). The *MMP12* −82A>G polymorphism has been associated with coronary atherosclerosis [15], [49], [50]. A study by Jormsjo et al. suggests this polymorphism is associated with narrowing of coronary arteries in diabetic patients with CHD [15], [50].

GenHAT is an ancillary study of ALLHAT. The ALLHAT study population included only those over age 55 with hypertension. As a result, our findings may not be generalizable to younger age groups. An additional limitation is that we looked at only four MMP polymorphisms. There may be unknown polymorphisms in these genes or combinations of polymorphisms affecting the findings. Since we performed multiple tests of pharmacogenetic effects, these findings would not meet the threshold of statistical significance if corrected for multiple testing (Bonferroni correction: 0.05/72 tests would equate to a *P*-value of 0.0007). However, given the possibility of linkage disequilibrium between variants and non-mutually exclusive outcomes, this threshold is overly conservative. Nevertheless, the likelihood that these findings are false positives is not negligible, and independent replication is necessary. At this time, there are no other large clinical trials that are outcome-based like ALLHAT in which results can be replicated. Our findings and the findings of other studies suggest that future studies might fruitfully investigate the functional interactions of MMP-9 and MMP-12 with diuretics, ACE inhibitors, and CCBs. This study has several strengths. The ALLHAT trial was a large, double-blind randomized trial. Additionally, the study population showed exceptional ethnic and gender diversity (about 50% non-White, about 50% female) [26]. Our results were not sensitive to departures from HWE in various ethnic groups.
